# The Association of a Lower Risk of Fibromyalgia with Human Papillomavirus Vaccination: A Retrospective Cohort Study from the TriNetX US Collaborative Network

**DOI:** 10.3390/vaccines13030235

**Published:** 2025-02-25

**Authors:** Lin-Hong Shi, An-Ping Huo, Shiow-Ing Wang, Pui-Ying Leong, James Cheng-Chung Wei

**Affiliations:** 1 JC School of Public Health and Primary Care, The Prince of Whales Hospital, The Chinese University of Hong Kong, New Territories, Hong Kong; jennyshi@link.cuhk.edu.hk; 2 Institute of Medicine, Chung Shan Medical University, No. 110, Sec. 1, Jianguo N. Rd., South District, Taichung City 40201, Taiwan; antonio.aphuo@msa.hinet.net (A.-P.H.); shiowing0107@gmail.com (S.-I.W.); 3 Division of Allergy, Immunology and Rheumatology, Department of Internal Medicine, Chung Shan Medical University Hospital, No. 110, Sec. 1, Jianguo N. Rd., South District, Taichung City 40201, Taiwan; 4 Center for Health Data Science, Department of Medical Research, Chung Shan Medical University Hospital, No. 110, Sec. 1, Jianguo N. Rd., South District, Taichung City 40201, Taiwan; 5 Department of Health Policy and Management, College of Health Care and Management, Chung Shan Medical University, No. 110, Sec. 1, Jianguo N. Rd., South District, Taichung City 40201, Taiwan; 6 School of Medicine, Chung Shan Medical University Hospital, No. 110, Sec. 1, Jianguo N. Rd., South District, Taichung City 40201, Taiwan; 7 PhD Program of Business, Feng Chia University, No. 100, Wenhua Rd, Xituan District, Taichung 407102, Taiwan; 8 Shanxi Bethune Hospital, Shanxi Academy of Medical Sciences, Third Hospital of Shanxi Medical University, Tongji Shanxi Hospital, Taiyuan 030032, China; 9 Department of Allergy, Immunology and Rheumatology, Chung Shan Medical University Hospital, No. 110, Sec. 1, Jianguo N. Rd., South District, Taichung City 40201, Taiwan; 10 Graduate Institute of Integrated Medicine, China Medical University, No. 100, Sec. 1, Jingmao Rd., Beitun Dist., Taichung City 406040, Taiwan; 11 Institute of Medicine/Department of Nursing, Chung Shan Medical University, No. 110, Sec. 1, Jianguo N. Rd., South District, Taichung City 40201, Taiwan

**Keywords:** human papillomavirus vaccination, HPV, vaccine, fibromyalgia, TriNetX, cohort

## Abstract

Objective: Remarkably similar symptoms have been observed between fibromyalgia patients and those who present adverse events after HPV vaccination. However, no research has been conducted on their association. Methods: Using data from the US collaborative network within the TriNetX network, we identified all the females who had had an HPV vaccination within 1 year before an index date falling between 2016 and 2023. We selected a propensity-score-matched (PSM, 1:1 ratio), non-HPV-vaccinated cohort as the comparator. Both cohorts were followed up from 1 day after the index date until the diagnosis of incidental fibromyalgia or until the patient was lost to the follow-up process or until the end of 2023. Results: After PSM, a total of 421,564 females in the HPV-vaccinated cohort and 421,564 females in the non-HPV-vaccinated cohort were included in the study. Significantly lower risks of developing fibromyalgia after 5 years’ follow-up were consistent in different models after adjusting for different covariates (adjusted hazard ratios [aHRs]: 0.505, 0.665, and 0.601). Also, significantly lower risks of incident fibromyalgia were identified across different follow-up periods, namely, 1 day to 1 year (HR: 0.464; 95% CI, 0.386–0.559), 1 day to 3 years (HR: 0.553; 95% CI, 0.494–0.618), 1 day to 5 years (HR: 0.601; 95% CI, 0.549–0.658), and 1 day to 7 years (HR: 0.636; 95% CI, 0.587–0.690). Conclusions: This study demonstrates that HPV vaccination significantly decreases the risk of developing incident fibromyalgia across different follow-up periods and subgroups. Our study suggests that HPV vaccination may potentially reduce the risk of developing fibromyalgia in female patients, which needs validation through studies of the mechanisms involved.

## 1. Introduction

Human papillomaviruses (HPVs) are small double-stranded DNA viruses and have been associated with several cancers and autoimmune diseases [1,2,3]. Prophylactic HPV vaccines are considered a safe and effective measure to reduce HPV infection and HPV-related complications [4,5] and provide long-term protection based on the robust immune memory mediated by memory B cells [6]. However, existing HPV vaccine-related safety concerns had previously been pointed out [7]. It was reported that the most common adverse events associated with the HPV vaccine were usually transient, without lasting harm, and injection-site-related [8]. One study showed mild to moderate adverse events, including pain (71.6%), redness (25.6%), and swelling (21.8%) [9]. But suspected severe/systematic adverse events due to the HPV vaccines have also been reported in several countries, including widespread pain, disabling fatigue, headache, and memory impairment [8,10]. This constellation of symptoms and signs after HPV vaccination had previously been labeled with the diagnosis of fibromyalgia [11]. Given the remarkably similar symptoms between fibromyalgia patients and those who presented systematic side effects after HPV vaccination, it opens the question of whether there is an association between fibromyalgia and the HPV vaccine. To the best of our knowledge, no research has been conducted to address this field.

It has been reported that innate and adaptive immune responses can be effectively primed by the virus-like particles (VLPs) contained in HPV vaccines, which can efficiently activate local antigen-presenting cells to induce antibody production via toll-like receptors [12,13]. Also, therapeutic HPV vaccines are used to stimulate cell-mediated immune responses to target and kill infected cells specifically [14]. On the other hand, extensive research over the past decade has investigated the immunological background of fibromyalgia [15,16,17]. The balance between pro- and anti-inflammatory cytokines is suggested to be disrupted in favor of pro-inflammatory cytokines, including tumor necrosis factor-a (TNF-a), interleukin-1 (IL-1), IL-6, and IL-8 [18,19]. The immunological alterations may consequently lead to an inflammatory state in fibromyalgia [16,17,20]. However, the pathogenesis of fibromyalgia (FM) remains unclear. The crosstalk among innate/adaptive immunity may lead to central neuroinflammation and central sensitization, which are closely connected to fibromyalgia [19]. Whether the innate and adaptive immune responses primed by the HPV vaccination can increase or decrease the risk of fibromyalgia remains unknown.

Given the remarkably similar symptoms between fibromyalgia patients and some people who received HPV vaccine, we hypothesized that HPV vaccination could influence the risk of fibromyalgia via the crosstalk between innate/adaptive immunity. Therefore, we conducted this matched cohort study to investigate the association between the HPV vaccine and fibromyalgia, utilizing a large, multicenter, electronic medical record (EMR) network.

## 2. Methods

### 2.1. Data Source and Ethics

We used the EMR from the US collaborative network, a subset of the TriNetX network, which includes data from 64 global healthcare organizations (HCOs) and comprises approximately 78 million patients in the USA, for the primary analysis. The TriNetX network contains the data of demographics, diagnoses (represented by the International Classification of Diseases, Tenth Revision, Clinical Modification [ICD-10-CM] codes), medications (represented by the codes in the Veterans Affairs National Formulary), procedures (based on ICD-10-Procedure Coding System [ICD-10-PCS] or Current Procedural Terminology [CPT]), healthcare utilization, and laboratory measurements (based on in Logical Observation Identifiers Names and Codes [LOINC]). TriNetX has demonstrated significant advantages for epidemiological studies [21,22,23]. Our study design was similar to that described in previous publications using the TriNetX platform [21], although with several modifications reflecting the longer duration of follow-up and the different questions being addressed.

The TriNetX analytics network is compliant with the Health Insurance Portability and Accountability Act (HIPAA) and the General Data Protection Regulation (GDPR) and was granted a waiver by the Western Institutional Review Board (WIRB) as it solely used aggregated counts and statistical summaries of de-identified data. The study protocol and survey instrument were approved by the Institutional Review Board of Chung Shan Medical University Hospital in Taiwan (CSMUH No: CS2-21176, date of approval: 1 December 2021). The reporting was informed by the REporting of studies Conducted using Observational Routinely-collected health Data (RECORD) Statement for cohort studies [24].

### 2.2. Study Design and Participants

For this retrospective cohort, the full study period spanned from 1 January 2016 to 31 December 2023, with the main analysis spanning from 1 January 2016 to 31 December 2021. The participants were divided into two cohorts based on whether or not HPV vaccination was documented in their EMR within the TriNetX network. The HPV-vaccinated cohort consisted of participants who had received a 2-valent, 4-valent, or 9-valent HPV vaccine. The HPV vaccination was identified based on the following codes: CVX (Vaccines Administered) 137, RXNORM code 798262, 798264, 798266, 798268, 1596930~1596934.

A contemporaneous propensity-score-matched, non-HPV-vaccinated cohort was constructed, comprising patients who had neither received the HPV vaccine(s) nor presented the HPV antigen (LOINC code 17399-7, 17401-1, 17411-0, 17412-8, 12223-4), as documented in the TriNetX network at any time. The risks of outcomes were assessed in terms of HPV vaccination within one year before the index date. The first visit during the study period was referred to as the index date for the non-HPV-vaccinated cohort, while the date to receive the first vaccination was referred to as the index date for the HPV-vaccinated cohort.

**Figure 1:**
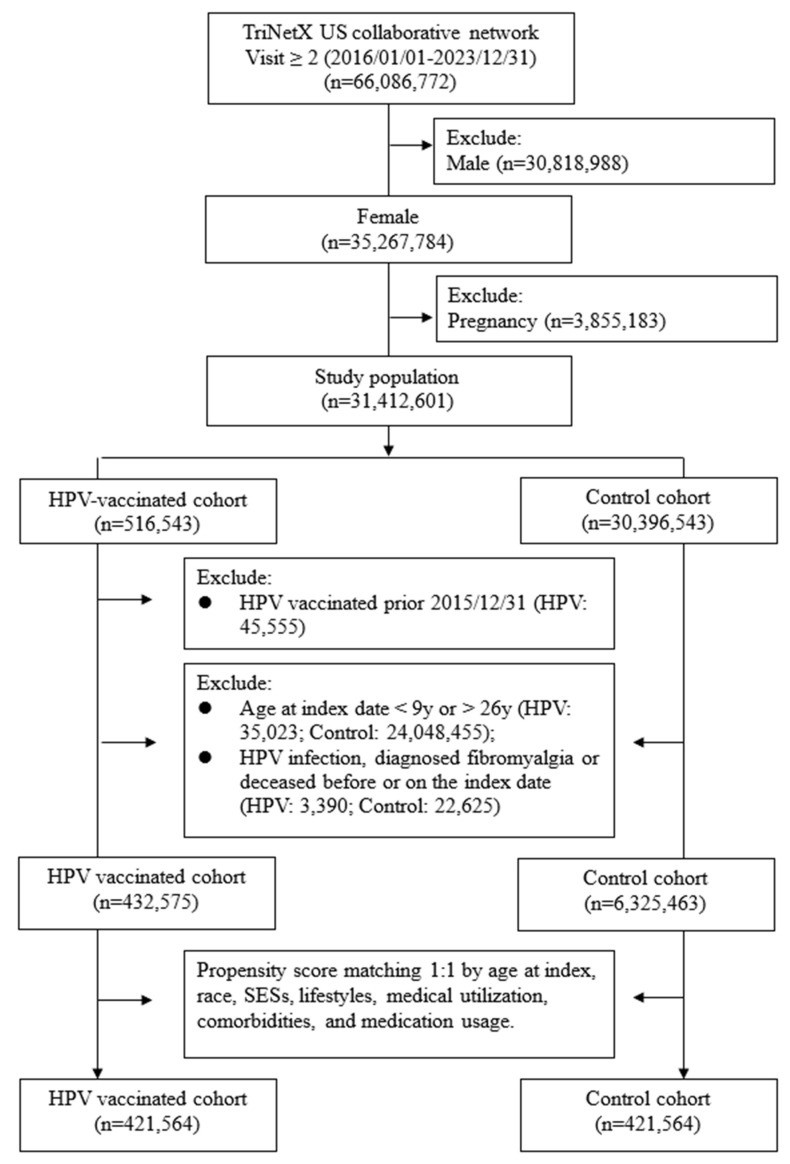
Flow chart of selection.

To reduce the detection bias, we only included female patients aged 9–26 years who were not pregnant (ICD-10 codes O00-O9A, Z33, Z34, Z3A; Lab code 82810; ICD-10-PCS codes beginning with 10) and who had at least two medical visits documented in their EMR within the TriNetX’s US collaborative network. We excluded female patients diagnosed with an HPV infection before or on the index date (ICD-10 codes R87.81, 87.82; B97.7), or who had received any dose of the HPV vaccine before 31 December 2015. The patients diagnosed with fibromyalgia or HPV-caused cancer (ICD-10 codes C51-C53) before or on the index date were also excluded. The flowchart for the cohort construction is depicted in Figure 1.

### 2.3. Outcomes

The index event (primary outcome) is referred to as the first diagnosis of fibromyalgia (ICD-10 CM code M79.7). Secondary outcomes were medical utilizations, such as hospital inpatient services, emergency department services, critical care services, and mechanical ventilation. Both cohorts were followed from one day after the index date up to five years later.

To further explore the effect of the vaccine on the risk of fibromyalgia in both cohorts with respect to different follow-up periods, we separately assessed the risk of fibromyalgia in terms of four different lengths of follow-up time: (1) one day to one year after the index date, (2) one day to three years after the index date, (3) one day to five years after the index date, and (4) one day to seven years after the index date.

### 2.4. Covariates

To adjust for the difference in baseline characteristics between the two cohorts, we incorporated the following covariates. To obtain the information on baseline characteristics, we traced the records within one year before the index date. Demographic covariates included the age at index, race, problems related to housing and economic circumstances (ICD-10 codes Z59, as a proxy for socioeconomic status), and problems related to education and literacy (ICD-10 codes Z55, as a proxy for economic status). Lifestyle-related covariates included nicotine and tobacco dependence (ICD-10 codes F17 and Z72.0, as a proxy for smoking) and alcoholic-related disorders (ICD10 codes F10, as a proxy for alcohol use). Baseline comorbidities included essential hypertension (I10), ischemic heart diseases (I20-25), cerebrovascular diseases (I60-69), obesity (E66), hyperlipidemia (E78.5), diabetes mellitus (DM, E08-13), vitamin D deficiency (E55), asthma (J45), chronic lower respiratory diseases (J40-47), chronic kidney disease (N18), systemic lupus erythematosus (SLE, M32), rheumatoid arthritis (RA, M05, M06), ankylosing spondylitis (AS, M45), psoriasis (L40), inflammatory diseases of female pelvic organs (N70-77), liver diseases (K70-77), periodontitis (K05), sleep disorders (G47), psychoactive substance use (F10-19), depression (F32), anxiety, traumatic past experiences, stress (F40-F48), blood diseases and disorders involving immune mechanisms (D50-D89), and neoplasm (C00-D49). Baseline medication use included corticosteroids for systemic use (Anatomical Therapeutic Chemical, ATC code H02), non-steroidal anti-inflammatory drugs (NSAIDs, ATC code M01A), oral contraceptives (G03A), and HMG-CoA reductase inhibitors (C10AA). Laboratory measurements, including the body mass index (BMI; obesity was defined as BMI ≥ 30 kg/m^2^), hemoglobin (<12 g/dL), and the presence of nuclear antibodies, rheumatoid factors, and human leukocyte antigen B27 (HLA-B27) were explored.

We tried our best to put the possible influencing factors mentioned in the literature into our analysis as covariates and perform PSM in an attempt to balance the baseline characteristics of the two groups; however, there may still have been unmeasured confounding factors, so we added a new E-value into the tables. The E-value is an indicator used to assess the extent to which unmeasured confounders might influence the study results. We have also included the E-value methodology. If E-values are greater than 2, it would require extremely strong unmeasured confounders to overturn the study conclusions, indicating the robustness of our findings.

### 2.5. Statistical Analysis

The TriNetX built-in function was used to conduct propensity score matching (PSM) at a 1:1 ratio by greedy nearest neighbor matching for age, sex, race, problems related to housing and economic circumstances (SESs), lifestyles, medical utilization, comorbidities, and medication usage between the two cohorts. The level of balance was evaluated for baseline characteristics between the propensity-score-matched cohorts by using standardized mean differences (SMDs), with any variable having an SMD < 0.1 being considered well-matched.

An adjusted hazard ratio (aHR) was used to quantify the relative risks of fibromyalgia based on the time-to-event analysis for the HPV-vaccinated and control groups. Cox proportional hazards models were performed to calculate the aHRs and the associated 95% confidence intervals (95% CI), along with the test for proportionality, using R’s Survival package v3.2-3. The proportional hazard assumption was tested using the generalized Schoenfeld approach built into the TriNetX network. The Kaplan–Meier method was used for the survival probability. The *p*-value threshold used in this study was 0.05. If the *p*-value was less than 0.05, the null hypothesis was rejected, indicating a statistically significant difference between the two groups. In order to assess the potential impact of unmeasured confounders on the study’s results, E-values were calculated using the observed hazard ratios (HRs) with a freely available online calculator (https://www.evalue-calculator.com/evalue/, accessed on 24 January 2025).

We further conducted subgroup analyses based on age (9–14 years and 15–26 years), race (White, Black/African American, Asian), comorbid obesity (defined by BMI ≥ 30 kg/m^2^ or diagnosed as being overweight or obese ICD-10 code E66), and comorbid depression (ICD-10-CM code F32-F33, with, without) to explore the differences between these subgroups in terms of the risk of incident fibromyalgia over a 5-year follow-up period, comparing HPV-vaccinated individuals and those who had never received an HPV vaccination.

To verify the robustness and consistency of our findings, we further performed a series of sensitivity analyses as follows: (1) using the same study design but with a different female group, aged 27–45 years; (2) replacing the control group with those who received the influenza vaccine to account for potential differences between participants who received the HPV vaccine and other vaccines; (3) addressing the potential immortal time bias for females allocated to the HPV-vaccinated cohort by modifying the study design to replace the index date of both cohorts with the date of the first visit [25]. These additional analyses were designed to ensure that our results were consistent and reliable across various scenarios and subpopulations.

## 3. Results

### 3.1. Baseline Characteristics

The baseline characteristics of the two cohorts are presented in Table 1. Before PSM, there was a disparity between the HPV-vaccinated cohort (n = 432,575) and the non-HPV-vaccinated cohort (n = 6,325,463) regarding various factors, including the age (mean age, 13.4 vs. 16.9 years), percentage of BMI ≥ 30 (2.9 vs. 0.9%), race (White [51.5 vs. 57.8%], Black or African American [21.5 vs. 14.9%], Asian [6.1 vs. 3.9%]), medical utilization (including office or other outpatient services [32.8 vs. 6.5%], preventive medicine services [12.8 vs. 2%], and emergency department services [5.0 vs. 1.6%]). The HPV-vaccinated cohort had a higher percentage of some medical comorbidities and medications. After PSM, the difference in most variables between the HPV-vaccinated cohort (n = 421,564) and non-HPV-vaccinated cohort (n = 421,564) was within the acceptable range (SMD < 0.1).

### 3.2. Risk of Fibromyalgia

As shown in Supplementary Table S1, the significant risk reduction of developing fibromyalgia after 5 years of follow-up was consistent across the different models, adjusting for different covariates (aHR: 0.505, 0.665, and 0.601, respectively). However, the risk was not significant when propensity score matching was performed on the age at index or race. Table 2 demonstrates the risks of incident fibromyalgia, comparing those who received HPV vaccination and those who did not, stratified by the different lengths of follow-up time after HPV vaccination.

**Figure 2:**
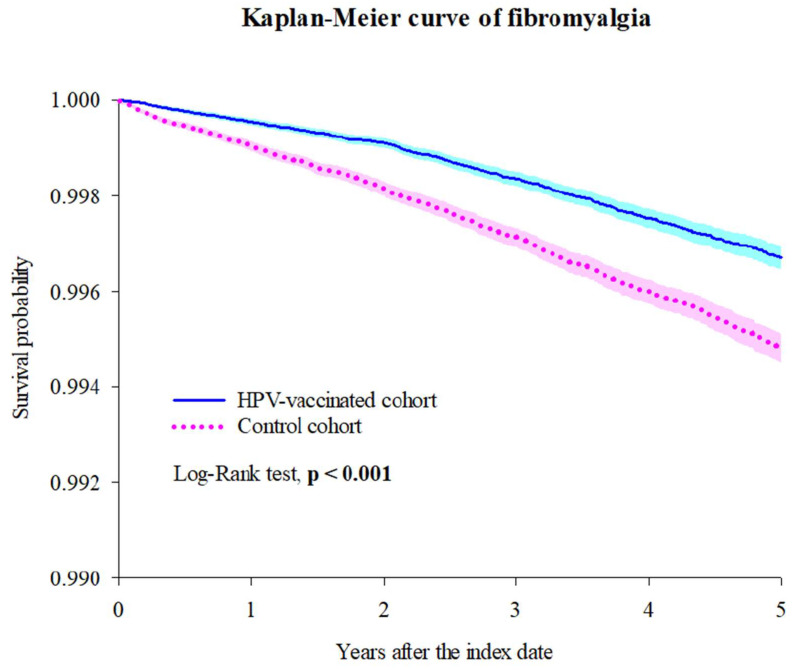
Kaplan–Meier curve of fibromyalgia.

We found significantly lower risks of incident fibromyalgia from 1 day after the index date to 1 year later among those who received HPV vaccination (HR: 0.464; 95% CI, 0.386–0.559). When the follow-up period was extended from 1 day to 3 years or 5 years or 7 years after the index date (Table 2 and Supplementary Table S2), significantly and consistently lower risks of incident fibromyalgia were found among those who received HPV vaccination: 1 day to 3 years (HR: 0.553; 95% CI, 0.494–0.618), 1 day to 5 years (HR: 0.601; 95% CI, 0.549–0.658), 1 day to 7 years (HR: 0.636; 95% CI, 0.587–0.690). In the Kaplan–Meier curves (Figure 2), significantly different risks of incident fibromyalgia were found between the HPV-vaccinated cohort and the control cohort during the 5-year follow-up period (log-rank test, *p* < 0.001).

### 3.3. Medical Utilization

In Supplementary Table S1, we found a consistent and significantly lower risk of fibromyalgia throughout the 5-year follow-up period among those who received HPV vaccination compared to those who did not in different models. This pattern was observed for hospital inpatient services (aHR: 0.944, 0.926, 0.884, and 0.871, respectively), emergency department services (aHR: 0.623, 0.600, 0.593, and 0.647, respectively), critical care services (aHR: 0.836, 0.706, 0.515 and 0.532, respectively), and mechanical ventilation (aHR: 0.604, 0.679, 0.461 and 0.463, respectively).

Furthermore, significantly lower risks of hospital inpatient services were observed for different follow-up periods—1 day to 1 year, 1 day to 3 years, 1 day to 5 years, and 1 day to 7 years (Supplementary Table S2).

### 3.4. Subgroup Analysis

#### 3.4.1. Age

**Figure 3:**
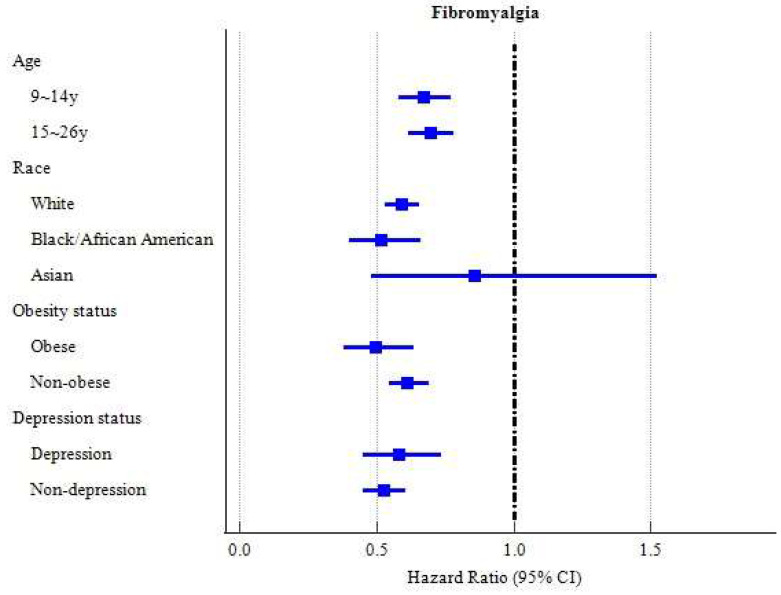
Forest of fibromyalgia (subgroup analyses).

We further examined the risk of fibromyalgia incidence in subgroups stratified by age (Supplementary Table S3 and Figure 3). Among the subgroups who were aged 9–14 years and aged 15–26 years, those who had received HPV vaccination had a lower risk of incident fibromyalgia across a 5-year follow-up period compared to their matched counterparts without HPV vaccination (HR: 0.670 and 0.695, respectively). Additionally, the hazard ratios of all types of medical utilization across a 5-year follow-up period appeared to be lower among the HPV cohort in all the age subgroups (Supplementary Table S3).

#### 3.4.2. Race

Among racial subgroups, those who had received HPV vaccination had a lower risk of incident fibromyalgia across a 5-year follow-up period when compared to the matched counterparts without HPV vaccination, with HRs of 0.589 for White and 0.515 for Black/African American, but not for Asian individuals (Supplementary Table S4 and Figure 3). Furthermore, the hazard ratios of incident fibromyalgia across the 5-year follow-up period appeared lower among the HPV cohort in all the racial subgroups (Supplementary Table S4).

#### 3.4.3. Obesity

A significant association between HPV vaccination and a lower risk of incident fibromyalgia across a 5-year follow-up period was found in subgroups with and without comorbid obesity, with HRs of 0.494 and 0.612, respectively (Supplementary Table S5 and Figure 3). Furthermore, the hazard ratios of all types of medical utilization across the 5-year follow-up period appeared to be lower among the HPV-vaccinated cohort in both obese and non-obese subgroups, except for inpatient use among obese subjects. The HPV-vaccinated cohort had an increased risk of hospital inpatient utilization (HR: 1.176; 95% CI: 1.129–1.226) compared to the control cohort. (Supplementary Table S5).

#### 3.4.4. Depression

HPV vaccination was associated with lower risks of incident fibromyalgia across a 5-year follow-up period among both subgroups with and without comorbid depression (Supplementary Table S6 and Figure 3). Furthermore, the hazard ratios of all types of medical utilization across the 5-year follow-up period appeared to be lower among the HPV-vaccinated cohort in all the subgroups, with and without depression (Supplementary Table S6).

### 3.5. Sensitivity Analysis

In the sensitivity analysis, we first applied the same study design to a different female group, aged 27–45 years, and found a similar pattern to the results from our main analysis (Supplementary Table S7). Secondly, we replaced the control group with those who received the influenza vaccine and discovered a consistent and significantly lower risk of incident fibromyalgia among the HPV-vaccinated cohort during the 5-year follow-up period (Supplementary Table S8). Finally, after modifying the study design to address the immortal time bias, we observed a similar pattern to the results from our main analysis (Supplementary Table S9).

To assess the potential confounding effect that might be caused by other vaccines administrated during the same period of time as the HPV vaccine, we performed an analysis excluding patients who received any other vaccines we could find in the database during the same study period. The decreased risk of fibromyalgia in the HPV-vaccinated females remained unchanged (Supplementary Table S10).

## 4. Discussion

In this large, multicenter cohort study using advanced analytics from the TriNetX network, we observed a negative association: women who received an HPV vaccination had a lower risk of incident fibromyalgia over a 5-year follow-up period. In terms of clinical outcomes, HPV vaccination was associated with a lower risk of multiple types of medical utilization. Furthermore, the reduced risks of incident fibromyalgia and medical utilizations were consistent across different follow-up periods after the index date.

In the absence of a clear etiology, biological, psychological, and social factors appear to contribute to fibromyalgia in patients. Consequently, treating fibromyalgia remains challenging, necessitating a shift in the philosophy of care from a biomedical model to a biopsychosocial model [26,27]. Our study suggests that HPV vaccination may potentially reduce the risk of developing fibromyalgia in female patients. This finding needs further validation through studies of mechanisms.

The HPV vaccine has been associated with the risk of autoimmune disease. For instance, a patient was reported to developed SLE after receiving a quadrivalent HPV vaccine [28], and eleven similar cases have been identified in the literature since 2006. Besides SLE, HPV vaccination has also been linked to other autoimmune diseases in genetically susceptible individuals, such as spondyloarthritis [29] and rheumatoid arthritis (RA) [29]. However, some robust population cohort studies disprove that there is an association [30]. Fibromyalgia is frequently considered as a comorbidity in several autoimmune diseases, including SLE [31], primary antiphospholipid syndrome (PAPS) [32], primary Sjögren’s syndrome (pSS) [33], RA [34], and psoriatic arthritis (PsA) [35]. Our findings indicate a negative correlation between HPV vaccination and the risk of incident fibromyalgia. This reverse association between HPV vaccination and fibromyalgia may be attributed to different mechanisms underlying the association between fibromyalgia and these autoimmune diseases.

Although our study observed the negative correlation between HPV vaccination and the risk of incident fibromyalgia, the definite mechanisms underlying this negative association remain unclear. There are three types of opioid receptors—mu-opioid, delta-opioid, and kappa-opioid receptors [36]—expressed by immune cells in humans, including B lymphocytes [37]. Research has demonstrated decreased mu-opioid receptor availability in fibromyalgia patients [38], suggesting a dysfunction of the endogenous analgesic mechanisms in these individuals. Another study indicated that reduced B lymphocytes expressing mu-opioid receptors is a specific biomarker for fibromyalgia patients [39].

Notably, the long-term protection conferred by the HPV vaccine is based on robust immune memory that is mediated by memory B cells [6]. Memory B cells are a type of immune cell that “remembers” previous encounters with pathogens like HPV, allowing the immune system to respond more effectively upon re-exposure [6]. This indicates a potential role of B cells in the development of fibromyalgia following HPV vaccination. The HPV vaccine may act as a mu-opioid receptor agonist. Mu-opioid receptors are proteins on the surface of B cells that interact with opioid molecules to modulate pain perception and other physiological processes. We hypothesize that increased mu-opioid receptor expression on B cells, triggered by the HPV vaccine acting as a mu-agonist, might lead to negatively regulated pain perception and other physiological processes and, consequently, the decreased risk of fibromyalgia. This hypothesis was supported by two clinical trials [40,41] involving low-dose naltrexone, a mu-opioid receptor agonist. These trials demonstrated that low-dose naltrexone was effective in treating fibromyalgia by improving a wide range of symptoms. This evidence suggests that manipulating the mu-opioid receptor system may have beneficial effects in fibromyalgia.

Although a series of cases from several countries reported that HPV vaccine recipients developed suspected severe side effects presenting with a cluster of symptoms previously diagnosed as fibromyalgia [10], a large Danish study found that females who experienced severe or systematic adverse events following HPV vaccination had more pre-immunization symptoms and healthcare-seeking patterns than the control group [42]. The presence of similar symptoms, coupled with the small number of cases observed, does not necessarily equate to the occurrence of a specific disease (i.e., fibromyalgia) and cannot answer the question of causality regarding the HPV vaccine. Our findings suggest that the lower medical utilization observed in the vaccinated group could reflect a reduced healthcare burden, potentially due to the alleviation of fibromyalgia-related symptoms. Moreover, we did not observe a higher risk of multiple medical utilizations in our study, unlike the situation suggested in the article about the imbalanced health-seeking behavior seen in the Danish study [42], in which the comorbidities were present at the time of vaccination or were illnesses triggered by the HPV vaccine in a vulnerable subpopulation. As discussed previously, there may be a personal susceptibility to developing other illnesses after HPV vaccination.

Nevertheless, our study indicates that integrating HPV vaccination into broader preventive health strategies may lead to a reduction in fibromyalgia incidence, as well as leading to cost-effectiveness in treating fibromyalgia, potential policy changes, targeted vaccination recommendations for at-risk populations (like chronic pain patients with multiple episodes), and integration with other preventive health services. This holistic approach would lower the disease burden, improve public health outcomes, and promote overall well-being.

### Strengths and Limitations

This study provides the first evidence suggesting that HPV vaccination may prevent the incidence of fibromyalgia. Despite the potential biases associated with registry databases, we utilized a comprehensive and dynamically updated database within the TriNetX network. This approach enabled us to examine a wide global population within contemporary timeframes. Additionally, the validity of the HPV vaccination and fibromyalgia diagnoses are reliable in this dataset. We carefully matched groups for baseline characteristics using PSM and employed rigorous methodological approaches, including subgroup and sensitivity analyses, to minimize the risk of residual confounding.

However, several limitations must be acknowledged. First, the observational and retrospective design of this study may have introduced biases in the case selection, testing, and follow-up. As this is a retrospective cohort study, we cannot infer causality from the observed association between HPV vaccination and the fibromyalgia risk. Future studies investigating these mechanisms should be proposed to strengthen our findings. Despite efforts to mitigate any potential selection bias through socioeconomic matching, the lack of individual lifestyle data remains a limitation. Second, the use of EMR data can be susceptible to under-, over-, or misdiagnosis, potentially leading to residual confounding in our study. Third, inpatient records do not contain data on time-series HPV antibody level data in vaccinated individuals, preventing us from associating antibody data with outcomes. Additionally, we could not track the HPV infection status of patients. Also, the database used in this study restricted the source population to patients with medical insurance and healthcare visits in the USA during the study period. Therefore, the generalizability of our conclusions may be limited. In addition, for any cases seeking medical treatment at HCOs other than TriNetX, the related information is not available. There would have been some loss of follow-up and misclassification. Our findings need to be validated in other populations or national data resources with different vaccination rates, healthcare systems, or demographics, as the TriNetX dataset contained patients from large academic medical institutions. Lastly, it is highly possible that the patients may have received other vaccines during same period of time as the HPV vaccine administration, which may have biased our results. To make our results more robust, we also carried out a sensitivity analysis (Table S10), excluding the patients who received any other vaccines we could find in the database during the same study period. The decreased risk of fibromyalgia in the HPV-vaccinated females remained unchanged. We acknowledge that possible incomplete vaccination records in the TriNetX network could have led to the under-reporting of HPV vaccination, potentially biasing the findings. Future studies can address strategies for minimizing such biases, including incorporating additional patient-reported outcomes or external validation using other datasets.

## 5. Conclusions

In conclusion, our study suggests that HPV vaccination is associated with a lower risk of incident fibromyalgia and reduced multiple medical utilization over a 5-year follow-up period. These effects were consistently evident across different follow-up periods. Our study suggests that HPV vaccination may potentially reduce the risk of developing fibromyalgia in female patients, which needs validation through studies of mechanisms.

## Figures and Tables

**Table 1:** Baseline characteristics of study subjects (before and after PSM matching).

Variables	Before PSM			After PSM		
	HPV Cohort (n = 432,575)	Control Cohort (n = 6,325,463)	SMD	HPV Cohort (n = 421,564)	Control Cohort (n = 421,564)	SMD
**Age at Index, y**						
Mean ± SD	13.4 ± 3.9	16.9 ± 5.5	**0.738**	13.5 ± 3.9	13.6 ± 4.2	0.044
**Race, n (%)**						
White	222,974 (51.5)	3,655,671 (57.8)	**0.126**	220,415 (52.3)	221,486 (52.5)	0.005
Black or African American	92,804 (21.5)	942,907 (14.9)	**0.170**	89,285 (21.2)	70,737 (16.8)	**0.112**
Other race	35,837 (8.3)	434,179 (6.9)	0.054	34,045 (8.1)	33,547 (8.0)	0.004
Asian	26,237 (6.1)	245,589 (3.9)	**0.101**	25,443 (6.0)	16,341 (3.9)	**0.100**
Unknown race	51,056 (11.8)	999,304 (15.8)	**0.116**	48,829 (11.6)	75,980 (18.0)	**0.182**
American Indian or Alaskan Native	2040 (0.5)	23,023 (0.4)	0.017	1955 (0.5)	1825 (0.4)	0.005
Native Hawaiian or other Pacific Islander	1627 (0.4)	24,790 (0.4)	0.003	1592 (0.4)	1648 (0.4)	0.002
**Social economic status, n (%)**						
Problems related to education and literacy	852 (0.2)	2220 (0.0)	0.048	781 (0.2)	759 (0.2)	0.001
Problems related to housing and economic circumstances	654 (0.2)	718 (0.0)	0.049	594 (0.1)	533 (0.1)	0.004
**Lifestyles, n (%)**						
Nicotine dependence	839 (0.2)	5485 (0.1)	0.029	818 (0.2)	1489 (0.4)	0.030
Alcohol-related disorders	278 (0.1)	1832 (0.0)	0.016	270 (0.1)	440 (0.1)	0.014
Tobacco use	296 (0.1)	1159 (0.0)	0.024	291 (0.1)	332 (0.1)	0.004
**Medical utilization, n (%)**						
Office or other outpatient services	141,836 (32.8)	411,972 (6.5)	**0.701**	130,849 (31.0)	133,012 (31.6)	0.011
Preventive medicine services	55,481 (12.8)	127,954 (2.0)	**0.421**	49,633 (11.8)	50,450 (12.0)	0.006
Emergency department services	21,611 (5.0)	103,195 (1.6)	**0.189**	20,785 (4.9)	19,204 (4.6)	0.018
Hospital inpatient and observation care services	2327 (0.5)	14,824 (0.2)	0.049	2238 (0.5)	5469 (1.3)	0.081
**Comorbidities, n (%)**						
Anxiety, dissociative, stress-related, somatoform, and other nonpsychotic mental disorders	22,484 (5.2)	68,555 (1.1)	**0.238**	21,459 (5.1)	28,462 (6.8)	0.070
Chronic lower respiratory diseases	21,677 (5.0)	66,191 (1.0)	**0.233**	21,154 (5.0)	21,067 (5.0)	0.001
Asthma	20,572 (4.8)	62,215 (1.0)	**0.227**	20,126 (4.8)	20,177 (4.8)	0.001
Noninflammatory disorders of female genital tract	16,099 (3.7)	56,331 (0.9)	**0.189**	15,627 (3.7)	14,850 (3.5)	0.010
Overweight and obese	13,046 (3.0)	37,627 (0.6)	**0.183**	12,814 (3.0)	15,718 (3.7)	0.038
Depressive episode	9282 (2.1)	27,419 (0.4)	**0.152**	8893 (2.1)	13,441 (3.2)	0.067
Bacterial and viral infectious agents	9116 (2.1)	21,496 (0.3)	**0.161**	8647 (2.1)	4832 (1.1)	0.072
Diseases of the blood and blood-forming organs and certain disorders involving the immune mechanism	6110 (1.4)	29,384 (0.5)	0.098	5850 (1.4)	8278 (2.0)	0.045
Sleep disorders	6003 (1.4)	26,807 (0.4)	**0.102**	5661 (1.3)	8251 (2.0)	0.048
Vitamin D deficiency	5140 (1.2)	13,783 (0.2)	**0.116**	4973 (1.2)	6696 (1.6)	0.035
Neoplasms	4434 (1.0)	29,428 (0.5)	0.065	4387 (1.0)	10,342 (2.5)	**0.108**
Inflammatory diseases of female pelvic organs	3086 (0.7)	11,593 (0.2)	0.079	2947 (0.7)	2724 (0.6)	0.006
Major depressive disorder, recurrent	2590 (0.6)	6780 (0.1)	0.083	2498 (0.6)	2463 (0.6)	0.001
Diabetes mellitus	1867 (0.4)	17,963 (0.3)	0.025	1858 (0.4)	5731 (1.4)	0.097
Mental and behavioral disorders due to substance use						
Essential (primary) hypertension	1444 (0.3)	7231 (0.1)	0.046	1380 (0.3)	2290 (0.5)	0.033
Hyperlipidemia, unspecified	1141 (0.3)	4492 (0.1)	0.047	1076 (0.3)	1530 (0.4)	0.019
Gingivitis and periodontal diseases	632 (0.1)	1207 (0.0)	0.044	610 (0.1)	250 (0.1)	0.027
Diseases of liver	635 (0.1)	3592 (0.1)	0.028	614 (0.1)	1054 (0.3)	0.023
Inflammatory polyarthropathies	577 (0.1)	5313 (0.1)	0.015	565 (0.1)	1329 (0.3)	0.038
Chronic kidney disease (CKD)	475 (0.1)	3076 (0.0)	0.022	460 (0.1)	891 (0.2)	0.026
Psoriasis	431 (0.1)	2408 (0.0)	0.023	414 (0.1)	657 (0.2)	0.016
Cerebrovascular diseases	247 (0.1)	2667 (0.0)	0.007	240 (0.1)	801 (0.2)	0.038
Spondylopathies	246 (0.1)	1837 (0.0)	0.013	244 (0.1)	491 (0.1)	0.020
Systemic lupus erythematosus (SLE)	189 (0.0)	1666 (0.0)	0.009	183 (0.0)	457 (0.1)	0.024
Viral hepatitis	106 (0.0)	791 (0.0)	0.009	103 (0.0)	170 (0.0)	0.009
Ischemic heart diseases	58 (0.0)	435 (0.0)	0.006	54 (0.0)	111 (0.0)	0.010
**Medications, n (%)**						
Corticosteroids for systemic use	29,257 (6.8)	148,322 (2.3)	**0.213**	27,652 (6.6)	32,809 (7.8)	0.047
NSAIDs	26,435 (6.1)	147,081 (2.3)	**0.189**	24,936 (5.9)	27,091 (6.4)	0.021
Hormonal contraceptives for systemic use	14,129 (3.3)	122,403 (1.9)	0.084	13,996 (3.3)	17,686 (4.2)	0.046
HMG CoA reductase inhibitors/statins	263 (0.1)	1984 (0.0)	0.014	261 (0.1)	384 (0.1)	0.011
**Laboratories, n (%)**						
BMI, ≧30 (kg/m^2^)	12,358 (2.9)	57,807 (0.9)	**0.143**	12,004 (2.8)	12,102 (2.9)	0.001
Hemoglobin, <12 (g/dL)	6985 (1.6)	40,245 (0.6)	0.093	6647 (1.6)	9081 (2.2)	0.043
Nuclear Ab [presence] in serum, plasma, or blood	174 (0.0)	1461 (0.0)	0.010	165 (0.0)	401 (0.1)	0.022
Rheumatoid factor in serum or plasma, ≧14 IU/mL	32 (0.0)	220 (0.0)	0.005	30 (0.0)	49 (0.0)	0.005
HLA-B27 [presence] by flow cytometry (FC)	10 (0.0)	19 (0.0)	0.006	10 (0.0)	10 (0.0)	0.000

Note: Bold font represents a standardized difference that was more than 0.1. If the patient is less or equal to 10, results show the count as 10. HPV: human papillomavirus. PSM: propensity score matching. SD: standard deviation. SMD: standardized mean difference. NSAIDs: anti-inflammatory and anti-rheumatic products, non-steroids. HMG CoA reductase inhibitors: 3-hydroxy-3-methyl-gutaryl-CoA reductase inhibitors. BMI: body mass index. HLA: human leukocyte antigen. Ab: antibodies. Propensity score matching was performed on age at index, race (White), problems related to housing and economic circumstances (proxy to social economic status), lifestyle variables (nicotine dependence (proxy to smoking)), medical utilization (including office or other outpatient services, preventive medicine services), comorbidities (overweight and obese, diabetes mellitus, vitamin D deficiency, asthma, depressive episode, anxiety, dissociative, stress-related, somatoform, and other nonpsychotic mental disorders, neoplasms, and medication usage (corticosteroids for systemic use).

**Table 2:** Risk of fibromyalgia.

Follow-Up	Patients with Outcome		Hazard Ratio (95% CI) ^a^	E-value for Point Estimate (E-Value for the CI)
	HPV Cohort (n = 421,564)	Control Cohort (n = 421,564)		
1 day to 1 year	169	328	**0.464 (0.386–0.559)**	3.73 (2.98)
1 day to 3 years	500	798	0.553 (0.494–0.618)	3.02 (2.62)
1 day to 5 years	779	1179	0.601 (0.549–0.658)	2.71 (2.41)
1 day to 7 years	974	1503	0.636 (0.587–0.690) *	2.52 (2.26)

Note: Main model (1 day to 1 year) after HPV vaccination is highlighted in bold. HPV: human papillomavirus. CI: confidence interval. If the patient is less or equal to 10, the results show the count as 10. ^a^ Propensity score matching was performed on age at index, race (White), problems related to housing and economic circumstances (proxy to social economic status), lifestyle variables (Nicotine dependence (proxy to smoking)), medical utilization (including office or other outpatient services, preventive medicine services), comorbidities (overweight and obesity, diabetes mellitus, vitamin D deficiency, asthma, depressive episode, anxiety, dissociative, stress-related, somatoform, and other nonpsychotic mental disorders, neoplasms, and medication usage (corticosteroids for systemic use). * Proportionality < 0.001. If E-values are greater than 2, it would require extremely strong unmeasured confounders to overturn the study conclusions, indicating the robustness of our findings.

## Data Availability

Data are contained within the article.

## Supplementary Materials

The following supporting information can be downloaded at: https://www.mdpi.com/article/10.3390/vaccines13030235/s1. Table S1: Risk of outcomes (1 day to 5 years) adjusted for different variables, Table S2: Risk of outcome with different follow up duration, Table S3: Subgroup analysis of the risk of outcome (1 day to 5 years) stratified by age at index, Table S4: Subgroup analysis of the risk of outcome (1 day to 5 years) stratified by race, Table S5: Subgroup analysis of the risk of outcome (1 day to 5 years) stratified by obesity status, Table S6: Subgroup analysis of the risk of outcome (1 day to 5 years) stratified by depression status, Table S7: Sensitive analysis of the risk of outcome (1 day to 5 years) by changing the study cases to ages 27 to 45, Table S8: Sensitive analysis of the risk of outcome (1 day to 5 years) by replacing the control group with subjects who received the influenza vaccine, Table S9: Sensitive analysis of the risk of outcome (1 day to 5 years) by modifying study design to deal with immortal time bias, and Table S10: Risk of outcome (1 day to 5 years)_ exclude patients received other vaccines during same study period.
