# Supplementary material for: The Association of a Lower Risk of Fibromyalgia with Human Papillomavirus Vaccination: A Retrospective Cohort Study from the TriNetX US Collaborative Network
Source: Vaccines (Basel). 2025 Feb 25;13(3):235. doi: 10.3390/vaccines13030235 (PMC11946275; doi:10.3390/vaccines13030235)
Supplement: Supplementary file 1 [file vaccines-13-00235-s001.zip › vaccines-3434820-supplementary.pdf]

Table S1. Risk of outcomes (1 day to 5 years), adjusted for different variables.

Outcomes (HPV-vaccinated cohort vs. control cohort)	Hazard ratio (95% CI)			
	Model 1[a]	Model 2[b]	Model 3[c]	Model 4[d]
Fibromyalgia	**0.505 (0.471-0.543)***	0.908 (0.820-1.006)	**0.665 (0.605-0.729)**	**0.601 (0.549-0.658)**
Medical utilization				
Hospital inpatient services	**0.944 (0.934-0.954)***	**0.926 (0.912-0.940)***	**0.884 (0.871-0.897)***	**0.871 (0.858-0.884)***
Emergency department services	**0.623 (0.618-0.628)***	**0.600 (0.594-0.606)***	**0.593 (0.587-0.599)***	**0.647 (0.640-0.653)***
Critical care services	**0.836 (0.806-0.866)***	**0.706 (0.674-0.740)***	**0.515 (0.493-0.539)***	**0.532 (0.509-0.556)***
Mechanical ventilation	**0.604 (0.559-0.653)***	**0.679 (0.613-0.753)***	**0.461 (0.419-0.507)***	**0.463 (0.421-0.510)***

Note: HPV: human papillomavirus. CI: confidence interval. NA: not available.

a. Crude: before matching.
b. Propensity score matching was performed on age at index and race.
c. Propensity score matching was performed on age at index, race, problems related to housing and economic circumstances (proxy to social economic status), problems related to education and literacy (proxy to social economic status), lifestyle variables (tobacco use, nicotine dependence (proxy to smoking), alcohol-related disorders (proxy to alcohol drinking), and medical utilization (including office or other outpatient services, preventive medicine services, emergency department services, and hospital inpatient services).
d. Propensity score matching was performed on age at index, race (White), problems related to housing and economic circumstances (proxy to social economic status), lifestyle variables (nicotine dependence (proxy to smoking)), medical utilization (including office or other outpatient services, preventive medicine services), comorbidities (overweight and obese, diabetes mellitus, vitamin D deficiency, asthma, depressive episode, anxiety, dissociative, stress-related, somatoform, and other nonpsychotic mental disorders, neoplasms, and medication usage (corticosteroids for systemic use).

* Proportionality < 0.001.

Table S2. Risk of outcomes with different follow-up durations.

Outcomes (HPV-vaccinated cohort vs. control cohort)	Hazard ratio (95% CI)[a]			
	1 day to 1 year	1 day to 3 years	1 day to 5 years	1 day to 7 years
Fibromyalgia	**0.464 (0.386-0.559)**	**0.553 (0.494-0.618)**	**0.601 (0.549-0.658)**	**0.636 (0.587-0.690)***
Medical utilization				
Hospital inpatient services	**0.535 (0.523-0.548)***	**0.744 (0.732-0.757)***	**0.871 (0.858-0.884)***	**0.894 (0.881-0.906)***
Emergency department services	**0.531 (0.523-0.540)***	**0.610 (0.603-0.618)***	**0.647 (0.640-0.653)***	**0.664 (0.658-0.671)***
Critical care services	**0.373 (0.346-0.403)***	**0.481 (0.458-0.507)***	**0.532 (0.509-0.556)***	**0.557 (0.534-0.580)***
Mechanical ventilation	**0.300 (0.253-0.356)***	**0.423 (0.378-0.473)***	**0.463 (0.421-0.510)***	**0.482 (0.442-0.527)***

Note:
HPV: human papillomavirus. CI: confidence interval. NA: not available.

a. Propensity score matching was performed on age at index, race (White), problems related to housing and economic circumstances (proxy to social economic status), lifestyle variables (nicotine dependence (proxy to smoking)), medical utilization (including office or other outpatient services, preventive medicine services), comorbidities (overweight and obese, diabetes mellitus, vitamin D deficiency, asthma, depressive episode, anxiety, dissociative, stress-related, somatoform, and other nonpsychotic mental disorders, neoplasms, and medication usage (corticosteroids for systemic use).

* Proportionality < 0.001.

Table S3. The subgroup analysis of the risks of outcomes (1 day to 5 years), stratified by age at index.

Outcomes (HPV-vaccinated cohort vs. control cohort)	Hazard ratio (95% CI)[a]	
	9~14y (n=292,063 pairs)	15~26y (n=129,032 pairs)
Fibromyalgia	**0.670 (0.581-0.772)**	**0.695 (0.617-0.784)**
Medical utilization		
Hospital inpatient services	**0.815 (0.800-0.830)***	**0.963 (0.938-0.990)***
Emergency department services	**0.603 (0.595-0.610)***	**0.694 (0.681-0.707)***
Critical care services	**0.516 (0.490-0.544)***	**0.660 (0.605-0.721)***
Mechanical ventilation	**0.475 (0.423-0.535)***	**0.539 (0.454-0.639)**

Note:
HPV: human papillomavirus. CI: confidence interval. NA: not available.

a. Propensity score matching was performed on age at index, race (White), problems related to housing and economic circumstances (proxy to social economic status), lifestyle variables (nicotine dependence (proxy to smoking)), medical utilization (including office or other outpatient services, preventive medicine services), comorbidities (overweight and obese, diabetes mellitus, vitamin D deficiency, asthma, depressive episode, anxiety, dissociative, stress-related, somatoform, and other nonpsychotic mental disorders, neoplasms, and medication usage (corticosteroids for systemic use).

* Proportionality < 0.001.

Table S4. The subgroup analysis of the risks of outcomes (1 day to 5 years), stratified by race.

Outcomes (HPV-vaccinated cohort vs. control cohort)	Hazard ratio (95% CI)[a]		
	White (n=216,806 pairs)	Black/ African American (n=86,683 pairs)	Asian (n=24,875 pairs)
Fibromyalgia	**0.589 (0.529-0.657)***	**0.515 (0.399-0.663)**	0.855 (0.480-1.524)
Medical utilization			
Hospital inpatient services	**0.905 (0.888-0.923)***	**0.939 (0.911-0.968)***	**0.508 (0.468-0.552)***
Emergency department services	**0.688 (0.678-0.698)***	**0.584 (0.573-0.596)***	**0.471 (0.446-0.497)***
Critical care services	**0.565 (0.532-0.601)***	**0.573 (0.528-0.623)***	**0.379 (0.299-0.481)**
Mechanical ventilation	**0.503 (0.442-0.572)***	**0.491 (0.407-0.593)***	**0.396 (0.233-0.672)**

Note:
HPV: human papillomavirus. CI: confidence interval. NA: not available.

a. Propensity score matching was performed on age at index, race (White), problems related to housing and economic circumstances (proxy to social economic status), lifestyle variables (nicotine dependence (proxy to smoking)), medical utilization (including office or other outpatient services, preventive medicine services), comorbidities (overweight and obese, diabetes mellitus, vitamin D deficiency, asthma, depressive episode, anxiety, dissociative, stress-related, somatoform, and other nonpsychotic mental disorders, neoplasms, and medication usage (corticosteroids for systemic use).

* Proportionality < 0.001.

Table S5. The subgroup analysis of the risks of outcomes (1 day to 5 years), stratified by obesity status.

Outcomes (HPV-vaccinated cohort vs. control cohort)	Hazard ratio (95% CI)[a]	
	Obese [b] (n=39,142 pairs)	Non-obese [c] (n=315,836 pairs)
Fibromyalgia	**0.494 (0.383-0.637)**	**0.614 (0.545-0.692)***
Medical utilization		
Hospital inpatient services	**1.176 (1.129-1.226)***	**0.830 (0.815-0.845)***
Emergency department services	**0.862 (0.837-0.888)***	**0.627 (0.619-0.635)***
Critical care services	**0.516 (0.451-0.592)**	**0.511 (0.484-0.541)***
Mechanical ventilation	**0.403 (0.304-0.532)**	**0.414 (0.366-0.467)***

Note:
HPV: human papillomavirus. CI: confidence interval. NA: not available.

a. Propensity score matching was performed on age at index, race (White), problems related to housing and economic circumstances (proxy to social economic status), lifestyle variables (nicotine dependence (proxy to smoking)), medical utilization (including office or other outpatient services, preventive medicine services), comorbidities (overweight and obese, diabetes mellitus, vitamin D deficiency, asthma, depressive episode, anxiety, dissociative, stress-related, somatoform, and other nonpsychotic mental disorders, neoplasms, and medication usage (corticosteroids for systemic use).
b. The diagnosis of obesity (ICD-10-CM code E66) or a BMI ≥30 kg/m² occurred within 1 year before or on the index date.
c. The medical record has never shown a diagnosis of obesity (ICD-10-CM code E66) or a record of a BMI of greater than or equal to 30 kg/m².

* Proportionality < 0.001.

Table S6. The subgroup analysis of the risks of outcomes (1 day to 5 years), stratified by depression status.

Outcomes (HPV-vaccinated cohort vs. control cohort)	Hazard ratio (95% CI)[a]	
	Depression [b] (n=15,836 pairs)	Non-depression [c] (n=334,589 pairs)
Fibromyalgia	**0.579 (0.453-0.739)**	**0.524 (0.453-0.606)***
Medical utilization		
Hospital inpatient services	**0.780 (0.739-0.823)***	**0.884 (0.868-0.901)***
Emergency department services	**0.909 (0.870-0.950)***	**0.609 (0.601-0.617)***
Critical care services	**0.546 (0.462-0.645)**	**0.501 (0.473-0.530)***
Mechanical ventilation	**0.468 (0.326-0.670)**	**0.459 (0.406-0.518)***

Note:

HPV: human papillomavirus. CI: confidence interval. NA: not available.

a. Propensity score matching was performed on age at index, race (White), problems related to housing and economic circumstances (proxy to social economic status), lifestyle variables (nicotine dependence (proxy to smoking)), medical utilization (including office or other outpatient services, preventive medicine services), comorbidities (overweight and obese, diabetes mellitus, vitamin D deficiency, asthma, depressive episode, anxiety, dissociative, stress-related, somatoform, and other nonpsychotic mental disorders, neoplasms, and medication usage (corticosteroids for systemic use).

b. Diagnosed depression (ICD-10-CM code F32~F33) within 1 year before or on the index date.

c. The medical record has never shown any diagnosis of depression (ICD-10-CM code F32~F33).

* Proportionality < 0.001.

Table S7. The sensitivity analysis of the risks of outcomes (1 day to 5 years) by changing the study cases to ages 27 to 45.

Outcomes	Patients with outcome		Hazard ratio (95%CI) [a]	E-value for point estimate (E-value for the CI)
	HPV-vaccinated cohort (n=27587)	Control cohort (n=27587)		
Fibromyalgia	180	506	**0.558 (0.469-0.664)***	2.98 (2.38)
Medical utilization				
Hospital inpatient services	1862	3588	**0.748 (0.704-0.790)***	2.01 (1.85)
Emergency department services	3400	6123	**0.771 (0.739-0.805)***	1.92 (1.79)
Critical care services	184	436	**0.737 (0.617-0.881)**	2.05 (1.53)
Mechanical ventilation	48	188	**0.419 (0.303-0.580)**	4.21 (2.84)

Note:
HPV: human papillomavirus. CI: confidence interval. NA: not available.
If the patient is less or equal to 10, the results show the count as 10.
a. Propensity score matching was performed on age at index, race (White), problems related to housing and economic circumstances (proxy to social economic status), lifestyle variables (nicotine dependence (proxy to smoking)), medical utilization (including office or other outpatient services, preventive medicine services), comorbidities (overweight and obese, diabetes mellitus, vitamin D deficiency, asthma, depressive episode, anxiety, dissociative, stress-related, somatoform, and other nonpsychotic mental disorders, neoplasms, and medication usage (corticosteroids for systemic use).
* Proportionality < 0.001.
If E-values are greater than 2, it would require extremely strong unmeasured confounders to overturn the study conclusions, indicating the robustness of our findings.

Table S8. The sensitivity analysis of the risks of outcomes (1 day to 5 years) by replacing the control group with subjects who received the influenza vaccine.

Outcomes	Patients with outcome		Hazard ratio (95%CI) [a]	E-value for point estimate (E-value for the CI)
	HPV-vaccinated cohort (n=205056)	Influenza cohort (n=205056)		
Fibromyalgia	506	612	**0.691 (0.614-0.777)**	2.25 (1.89)
Medical utilization				
Hospital inpatient services	18117	17699	**0.868 (0.850-0.886)***	1.57 (1.51)
Emergency department services	33530	33561	**0.843 (0.830-0.856)***	1.66 (1.61)
Critical care services	1688	2328	**0.616 (0.578-0.656)***	2.63 (2.42)
Mechanical ventilation	366	707	**0.444 (0.391-0.503)**	3.93 (3.39)

Note:
HPV: human papillomavirus. CI: confidence interval. NA: not available.
If the patient is less or equal to 10, the results show the count as 10.
a. Propensity score matching was performed on age at index, race (White), problems related to housing and economic circumstances (proxy to social economic status), lifestyle variables (nicotine dependence (proxy to smoking)), medical utilization (including office or other outpatient services, preventive medicine services), comorbidities (overweight and obese, diabetes mellitus, vitamin D deficiency, asthma, depressive episode, anxiety, dissociative, stress-related, somatoform, and other nonpsychotic mental disorders, neoplasms, and medication usage (corticosteroids for systemic use).
* Proportionality < 0.001.
If E-values are greater than 2, it would require extremely strong unmeasured confounders to overturn the study conclusions, indicating the robustness of our findings.

Table S9. The sensitivity analysis of the risks of outcomes (1 day to 5 years) by modifying the study design to deal with an immortal time bias.

Outcomes	Patients with outcome		Hazard ratio (95%CI) [a]	E-value for point estimate (E-value for the CI)
	HPV-vaccinated c ohort (n=64319)	Control cohort (n=64319)		
Fibromyalgia	115	125	**0.632 (0.490-0.814)**	2.54 (1.76)
Medical utilization				
Hospital inpatient services	5005	4651	**0.788 (0.757-0.820)***	1.85 (1.74)
Emergency department services	9401	12115	**0.544 (0.530-0.559)***	3.08 (2.98)
Critical care services	478	541	**0.649 (0.574-0.735)**	2.45 (2.06)
Mechanical ventilation	94	117	**0.610 (0.464-0.801)**	2.66 (1.81)

Note:
HPV: human papillomavirus. CI: confidence interval. NA: not available.
If the patient is less or equal to 10, the results show the count as 10.

a. Propensity score matching was performed on age at index, race (White), problems related to housing and economic circumstances (proxy to social economic status), lifestyle variables (nicotine dependence (proxy to smoking)), medical utilization (including office or other outpatient services, preventive medicine services), comorbidities (overweight and obese, diabetes mellitus, vitamin D deficiency, asthma, depressive episode, anxiety, dissociative, stress-related, somatoform, and other nonpsychotic mental disorders, neoplasms, and medication usage (corticosteroids for systemic use).

* Proportionality < 0.001.
If E-values are greater than 2, it would require extremely strong unmeasured confounders to overturn the study conclusions, indicating the robustness of our findings.

Table S10. Risk of outcome (1 day to 5 years), excluding patients received other vaccines during same study period.

Outcomes	Patients with outcome		Hazard ratio (95%CI) [a]	E-value for point estimate (E-value for the CI)
	HPV-vaccinated cohort (n=29236)	Control cohort (n=29236)		
Fibromyalgia	50	129	**0.428 (0.309-0.594)**	4.10 (2.76)
Medical utilization				
Hospital inpatient services	2027	2434	**0.888 (0.837-0.942)***	1.50 (1.32)
Emergency department services	3848	5170	**0.786 (0.753-0.819)***	1.86 (1.74)
Critical care services	144	234	**0.666 (0.541-0.819)***	2.37 (1.74)
Mechanical ventilation	19	57	**0.362 (0.215-0.609)**	4.97 (2.67)

Note:
HPV: human papillomavirus. CI: confidence interval. NA: not available.
If the patient is less or equal to 10, the results show the count as 10.

a. Propensity score matching was performed on age at index, race (White), problems related to housing and economic circumstances (proxy to social economic status), lifestyle variables (nicotine dependence (proxy to smoking)), medical utilization (including office or other outpatient services, preventive medicine services), comorbidities (overweight and obese, diabetes mellitus, vitamin D deficiency, asthma, depressive episode, anxiety, dissociative, stress-related, somatoform, and other nonpsychotic mental disorders, neoplasms, and medication usage (corticosteroids for systemic use).

* Proportionality < 0.001
If E-values are greater than 2, it would require extremely strong unmeasured confounders to overturn the study conclusions, indicating the robustness of our findings.
